# Supplementary material for: LDH-A Promotes Metabolic Rewiring in Leucocytes from the Intestine of Rats Treated with TNBS
Source: Metabolites. 2023 Jul 12;13(7):843. doi: 10.3390/metabo13070843 (PMC10384056; doi:10.3390/metabo13070843)
Supplement: Supplementary file 1 [file metabolites-13-00843-s001.zip › Supplementary Table I.pdf]

Table 1: DAI score parameters. Weight loss, stool consistence and blood in stool in EtOH and TNBS groups.

Parameter	EtOH			TNBS		
	Previous Day	Later Day	Sacrifice Day	Previous Day	Later Day	Sacrifice Day
Weight loss	0 pts	0 pts	2 pts (5%)	0 pts	2 pts (5%)	2 pts (9%)
stool consistency	0 pts	0 pts	0 pts	0 pts	0 pts	2 pts
Presence of blood in stool	0 pts	0 pts	0 pts	0 pts	0 pts	0 pts
Total	0 pts	0 pts	2 pts	0 pts	2 pts	4 pts
