# Supplementary material for: LDH-A Promotes Metabolic Rewiring in Leucocytes from the Intestine of Rats Treated with TNBS
Source: Metabolites. 2023 Jul 12;13(7):843. doi: 10.3390/metabo13070843 (PMC10384056; doi:10.3390/metabo13070843)
Supplement: Supplementary file 1 [file metabolites-13-00843-s001.zip › Supplementary Table II.pdf]

Table 2. Initial activity in epithelial cells of EtOH and TNBS groups. Epithelial cell samples from the EtOH and TNBS groups. Epithelial cells of the TNBS group showed a significant increase in SDH enzyme activity ($p<0.05$). Data represent the mean ± SD of three independent experiments ($n = 6$). P-values were determined by the one-way ANOVA (** $p<0.0001$).

Epithelial Cells			
	Initial Activity		Statistical Difference
	EtOH	TNBS	
G6PDH	0.014 ± (0.001)	0.014 ± (0.0007)	p≤0.97
GAPDH	0.215 ± (0.025)	0.470 ± (0.286)	p≤0.19
LDH	0.641 ± (0.0107)	0.999 ± (0.074)	p≤0.63
IDH	0.117 ± (0.081)	0.187 ± (0.055)	p≤0.285
GDH	0.165 ± (0.097)	0.310 ± (0.049)	p≤0.083
SDH	0.011 ± (0.0001)	0.016 ± (0.0005)	**p≤0.0001**
